# A semi-supervised approach for rapidly creating clinical biomarker phenotypes in the UK Biobank using different primary care EHR and clinical terminology systems

**DOI:** 10.1093/jamiaopen/ooaa047

**Published:** 2020-12-05

**Authors:** Spiros Denaxas, Anoop D Shah, Bilal A Mateen, Valerie Kuan, Jennifer K Quint, Natalie Fitzpatrick, Ana Torralbo, Ghazaleh Fatemifar, Harry Hemingway

**Affiliations:** 1 Institute of Health Informatics, University College London, London, UK; 2 Health Data Research UK, University College London, London, UK; 3 The Alan Turing Institute, London UK; 4 British Heart Foundation Research Accelerator, University College London, London, UK; 5 King’s College Hospital, London, UK; 6 Institute of Cardiovascular Science, University College London, London, UK; 7 National Heart and Lung Institute, Imperial College London, London, UK

**Keywords:** electronic health records, phenotyping, medical informatics, UK Biobank

## Abstract

**Objectives:**

The UK Biobank (UKB) is making primary care electronic health records (EHRs) for 500 000 participants available for COVID-19-related research. Data are extracted from four sources, recorded using five clinical terminologies and stored in different schemas. The aims of our research were to: (a) develop a semi-supervised approach for bootstrapping EHR phenotyping algorithms in UKB EHR, and (b) to evaluate our approach by implementing and evaluating phenotypes for 31 common biomarkers.

**Materials and Methods:**

We describe an algorithmic approach to phenotyping biomarkers in primary care EHR involving (a) bootstrapping definitions using existing phenotypes, (b) excluding generic, rare, or semantically distant terms, (c) forward-mapping terminology terms, (d) expert review, and (e) data extraction. We evaluated the phenotypes by assessing the ability to reproduce known epidemiological associations with all-cause mortality using Cox proportional hazards models.

**Results:**

We created and evaluated phenotyping algorithms for 31 biomarkers many of which are directly related to COVID-19 complications, for example diabetes, cardiovascular disease, respiratory disease. Our algorithm identified 1651 Read v2 and Clinical Terms Version 3 terms and automatically excluded 1228 terms. Clinical review excluded 103 terms and included 44 terms, resulting in 364 terms for data extraction (sensitivity 0.89, specificity 0.92). We extracted 38 190 682 events and identified 220 978 participants with at least one biomarker measured.

**Discussion and conclusion:**

Bootstrapping phenotyping algorithms from similar EHR can potentially address pre-existing methodological concerns that undermine the outputs of biomarker discovery pipelines and provide research-quality phenotyping algorithms.

**LAY SUMMARY**

The UK Biobank study has collected extensive health-related information on participants such as what diseases they have been diagnosed with, what medications they have been taking, lifestyle risk factors (such as smoking or alcohol consumption) and other important measurements (such as blood pressure, body mass index, and glycated hemoglobin). Additionally, electronic health records (EHRs), data collected during visits to primary care physicians, will be shortly be made available for research purposes as they are a valuable resource of health information over longer periods of time. Researchers working with EHR data however face significant challenges as the data are extracted from four different sources, are recorded using different methods and are messy since they are not primarily collected for research but for care. Our research focused on creating a series of computer algorithms, which are used to extract important measurements related to participant’s health from EHR data in an efficient and accurate manner. We provide algorithms for 31 measurements (such as blood pressure, heart rate, weight, and cholesterol) which are commonly measured and used in primary care and evaluate them by examining their statistical distributions and their associated risk with death. These algorithms can be used by researchers to improve health and healthcare.

## INTRODUCTION

UK Biobank (UKB) is the largest longitudinal research study in the UK (∼500 000 participants), and one of the largest globally.^1^ To further enrich this cohort’s data, UKB has begun to link the wealth of information already collected from each individual to their primary care electronic health record (EHR).^1^ In the UK, the first point of contact with the health service for individuals with a new (non-emergency) medical problem or a chronic condition is their local general practitioner (GP). These GPs also receive information from the specialist health services that they refer their patients to, resulting in a closed loop communication system which should result in a complete (time-stamped) summary of their patients’ medical conditions, investigations, regular (prescribed) medications, etc. Introducing primary care EHR information will enable UKB researchers and policymakers to assess the course and outcomes of a plethora of different diseases and risk-factors at scale, whilst allowing them to simultaneously explore the genetic factors associated with each.

Prior to being able to interrogate the data for the ∼220 000 UKB participants that have already had their data linked, there is the non-trivial task of processing it such that it can be meaningfully interpreted. The primary care data that has been linked to the UKB are derived from the three different countries that compose the UK (England, Scotland and Wales). A total of four data sources (two in England, one in Scotland and one in Wales) using four different controlled clinical terminologies (containing more than 500 000 terms to record information) and different data schemas are used. As a result, researchers with no previous experience working with primary care EHR would need to dedicate a significant amount of time and effort to create phenotyping algorithms for important biomarkers, for example blood pressure or hematological laboratory markers. In this manuscript, we use the term “biomarker” to refer to well-established, and measurable, clinical or laboratory parameters that are used in routine clinical care as indicators of a particular disease or other physiological state. The challenge of poor methodological reproducibility in the biomarker discovery pipeline which lead to significant amounts of research waste are directly comparable to the challenges researchers face due to poor reproducibility of research findings due to the lack of consistent and replicable phenotyping approaches.^2–4^ Preventing waste in EHR-based biomarker research requires robust clinical validation of phenotypes. However, relying on individual clinical-academics to manually review and refine all of the phenotyping algorithms under development is not easily scalable. As such, an automated but more robust approach for creating and evaluating EHR phenotyping algorithms for biomarkers in primary care data is necessary to address the aforementioned methodological concerns.

One of the primary audiences of this research are US investigators since two-thirds of registered UKB investigators are from US-based institutions. Additionally, the controlled clinical terminologies used in UK EHR data are applicable to US data sources given that CTV3 is a subset of SNOMED-CT. Finally, these challenges are likely not unique to the UK Biobank nor to UK data but exist in other large-scale data resources, for example US initiatives such as Electronic Medical Records and Genomics (eMERGE),^5^ BioVU,^6^ Million Veteran Program,^7^ and All Of Us,^8^ where primary care EHR data is or will be ingested from multiple disparate data sources and requires significant amount of effort and pre-processing prior to statistical analysis.

The issues of scalability and methodological robustness have become even more relevant in light of UKB announcing that they will be making available the results of COVID-19 tests for participants through Public Health England (PHE), as well as a host of other relevant information, including intensive care data for those who test positive.^9^^,^^10^ It is likely that many non-EHR-specialists will be working UKB data for the foreseeable future, and given that the pandemic has already had significant and widespread societal, economic, medical, and health service impacts globally,^8^ there is an impetus to ensure rapid access of these critical data to researchers during this public health emergency, whilst ensuring that this does not come at the cost of research quality.^11^ The biomarkers we selected to examine and phenotype in this manuscript are all directly related to modifiable and non-modifiable risk factors for COVID-19 such as diabetes, blood pressure/hypertension, body mass index and obesity, cancer, and chronic obstructive pulmonary disease.^12–19^

## AIMS

The aims of the research presented in this article are two-fold:

- To describe a semi-supervised algorithm for rapidly bootstrapping EHR phenotyping algorithms for primary care data by UKB participants; and
- To provide phenotyping algorithms, metadata, implementation details, and validation evidence for 31 common biomarkers.

## METHODS

### Data sources

#### UK Biobank

Biomarker data for phenotyping were extracted from the UKB, a research study of ∼500 000 adults with extensive phenotypic and genotypic information. Currently, ∼44% of the cohort (*n* = 221 446) have data from primary care EHR linked and made available for researchers (Table 1). Data are collected from English, Scottish, and Welsh GP practices that make use of the EMIS (https://www.emishealth.com/), Vision (https://www.visionhealth.co.uk/), or TPP (https://www.tpp-uk.com/) primary care information systems. Data are recorded using four different controlled clinical terminologies: (1) Read version 2 (Read v2); (2) Clinical Terms Version 3 (CTV3); (3) British National Formulary (BNF); and (4) the Dictionary of Medicines and Devices (DM+D). Both Read v2 and CTV3 are part of the Systematized Nomenclature of Medicine Clinical Terms (SNOMED-CT)^20^ and since 2018 primary care practices in the UK are migrating to using SNOMED-CT terms exclusively.

**Table 1.** Primary care electronic health record data made available on UK Biobank participants

Country	Data source	Controlled clinical terminologies: clinical observations	Controlled clinical terminologies: prescriptions	Patients (*n*)	Clinical events (*n*)	Prescription events (*n*)	Data fields
England	Vision	Read v2	Read v2 DM+D	17 860	11 973 249	6 350 259	2
Scotland	EMIS, Vision	Read v2	BNF	26 269	11 365 300	4 301 151	3
England	TPP	CTV3	BNF	158 894	87 493 722	39 515 266	1
Wales	EMIS, Vision	Read v2	Read v2	20 463	12 837 100	7 533 324	2

*Note*: The number of patients reported was extracted from the registrations table and includes patients with more or one unique registration periods.

BNF: British National Formulary; CTV3: Clinical Terms Version 3; DM+D: Dictionary of Medicines and Devices; EMIS: Egton Medical Information Systems; TPP: The Phoenix Partners.

#### CALIBER

To enable the initial rapid prototyping of phenotype definitions for the biomarkers of interest, we used information from the CALIBER EHR resource and existing phenotyping algorithms derived using data from the Clinical Practice Research Datalink (CPRD). The resource has been described in detail elsewhere.^3^ In brief, the CALIBER resource provides reproducible phenotyping algorithms for linked EHR data based on three national sources: (a) longitudinal primary care data from the CPRD, (b) admitted patient care information on diagnoses and procedures from the Hospital Episode Statistics dataset, and (c) cause-specific mortality and socioeconomic deprivation information made available from the Office for National Statistics (ONS).

Primary care data were sourced from the general practices that submit data to the CPRD use the aforementioned Vision software (known as CPRD GOLD), and data are recorded using the Read version 2 clinical terminology system (containing 101 953 terms). In Vision EHR systems, the definition of the data columns is specified by the category of data in the record or the information archetype, which is called an ‘entity type’. For example, the blood pressure entity type specifies that the first structured data column (*value1*) is the diastolic and the second (*value2*) contains the systolic blood pressure. The associated Read v2 term may contain more details about the subtype of the measurement, for example ‘Standing blood pressure reading’.

### Biomarker data and unit recording

In the UKB, measurements from clinical observations (eg, blood pressure) or laboratory tests (eg, HbA1c) are recorded with a Read v2 or CTV3 term and up to three structured data columns (value1, value2, and value3). Each data provider uses a varying number of fields to capture information. For example, TPP in England uses a single value, with no explicit or implicit recording of units. Whereas, Scottish data sources are based on three fields, where the second data column (*value 2*) contains the units as free text. Vision-based systems are again different, as the units for recorded values are provided by a separate lookup file and are identified by a unique numeric code. To collate this information, the semi-supervised approach described below captures the relevant unit information across these different structures and processes them into consistent expressions utilizing mapping files to translate inconsistencies to the preferred unit type, for example ‘*×10^9/L*’, ‘*10^9/L*’, ‘*×10^9/L*’ map to ‘*10^9/L*’. Finally, these results were mapped to the Units of Measurement ontology^21^ (https://www.ebi.ac.uk/ols/ontologies/uo) using a Python script and manually reviewed such that any mismatches could be corrected.

### Semi-supervised phenotyping algorithm bootstrapping

**Figure 1.**
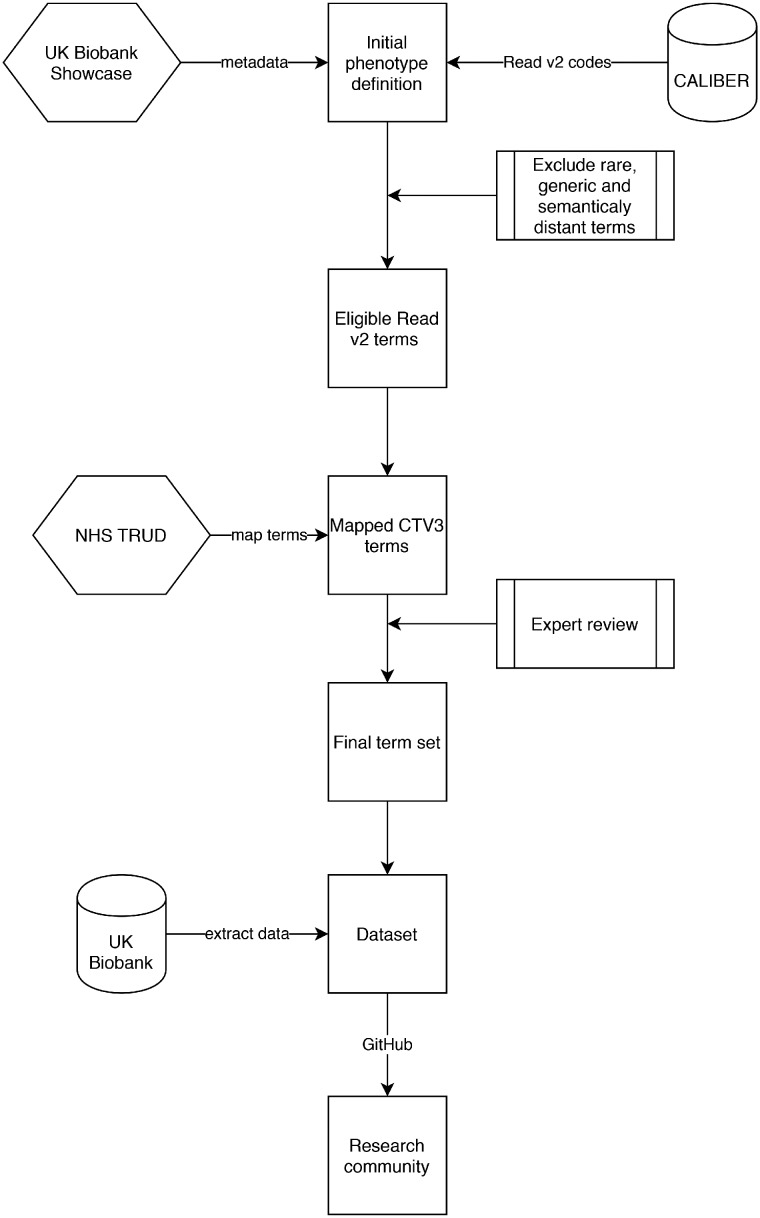
Description of main steps involved in the semi-supervised approach for rapidly creating electronic health record phenotyping algorithms for biomarkers in the UK Biobank. The main steps involved in the semi-supervised phenotyping process are: (1) seeding the algorithm definitions using existing phenotype algorithms from the CALIBER resource, (2) excluding generic, rare or semantically distant terms, (3) map Read version 2 terms to Clinical Terms Version 3 terms using the maps provided by the National Health Service (NHS) terminology service (TRUD), (4) expert review and manual inclusion/exclusion of terms, and (5) translation to SQL code and data extraction.

We identified 31 biomarkers spanning blood counts, clinical biochemistry results, and physical measurements based on their presumed importance with regards to modeling outcomes for COVID-19,^22^ as well as other more generic pathologies such as cardiovascular disease,^23^ and their availability (recorded at least once during the baseline assessment) (Table 2). In Vision EHR data, groups of similar clinical measurement types or laboratory tests have the same entity type. We used the entity type to identify candidate Read v2 terms which might identify equivalent data items in other data sources. For each biomarker, we performed the following process (Figure 1):

**Table 2.** Details on the 31 biomarkers used in this study spanning blood biochemistry, blood count and physical measures

Phenotype	UK Biobank field id	Phenotype type	Units	UnitOntology
ALP	30610	Blood biochemistry	U/L	UO_0000179
ALT	30620	Blood biochemistry	U/L	UO_0000179
Albumin	30600	Blood biochemistry	g/L	UO_0000175
CRP	30710	Blood biochemistry	mg/L	UO_0000273
Calcium	30680	Blood biochemistry	mmol/L	UO_0010003
Cholesterol	30690	Blood biochemistry	mmol/L	UO_0010003
Creatinine	30700	Blood biochemistry	umol/L	UO_0010003
Glucose	30740	Blood biochemistry	mmol/L	UO_0010003
HDL	30760	Blood biochemistry	mmol/L	UO_0010003
HbA1c	30750	Blood biochemistry	mmol/mol	UO_0010048
Total bilirubin	30840	Blood biochemistry	umol/L	UO_0010003
Triglycerides	30870	Blood biochemistry	mmol/L	UO_0010003
Urea	30670	Blood biochemistry	mmol/L	UO_0010003
Basophills	30160	Blood count	10^9/L	UO_0000317
Eosinophills	30150	Blood count	10^9/L	UO_0000317
Hematocrit perc	30030	Blood count	%	UO_0000187
Hemoglobin conc	30020	Blood count	g/dL	UO_0000208
Lymphocytes	30120	Blood count	10^9/L	UO_0000317
MCHb conc	30060	Blood count	g/dL	UO_0000208
MCV	30040	Blood count	fL	UO_0000104
Monocytes	30130	Blood count	10^9/L	UO_0000317
Neutrophils	30140	Blood count	10^9/L	UO_0000317
Platelets	30080	Blood count	10^9/L	UO_0000317
RBC	30010	Blood count	10^12/L	UO_0000317
WBC	30000	Blood count	10^9/L	UO_0000317
DBP	4079	Physical measures	mmHg	UO_0000272
FEV1	3063	Physical measures	L	UO_0000099
FVC	3062	Physical measures	L	UO_0000099
Height	50	Physical measures	cm	UO_0000015
SBP	4080	Physical measures	mmHg	UO_0000272
Weight	21002	Physical measures	Kg	UO_0000009

*Note*: For units, we provide the UnitOntology entry identifier. The UK Biobank field id column provides the field identifier for the respective biomarker measure, if available, derived from the research data collected at baseline.

ALP: alanine aminotransferase level; ALP: alkaline phosphatase level; CRP: C-reactive protein; DBP: diastolic blood pressure; FEV1: forced expiratory volume in 1 second; FVC: full vital capacity; HDL: high-density lipoprotein; MChb conc: mean corpuscular hemoglobin concentration; MCV: mean corpuscular volume; RBC: red blood cell; SBP: systolic blood pressure; WBC: white blood cell.

1. We manually mapped UKB fields to Vision entity type identifiers, for example for lymphocyte counts, the UKB field id is *30210* and the Vision entity type is *208*. We extracted from the UKB showcase information on the units, minimum and maximum value range and mean and identified any relevant unit conversions required.
2. For each Vision entity type, we generated a list of Read v2 terms used to record that biomarker and their frequency. We extracted the Read v2 term with the highest frequency (defined as the “*accepted term*”), for example for lymphocytes the term “*42M.00 Lymphocyte count*” was the term most used to record the lab values.
3. We applied a series of automated consistency checks to reduce the number of terms requiring manual review by domain experts. Specifically:
  - We excluded terms that were rarely used, that is occurring less than 1000 times and generic Read codes which did not specify the type of biomarker measured, for example “*4….00 Laboratory procedures*”.
  - For lipid measurements, we excluded plasma-based measurements and retained serum-derived values. We allowed pre-treatment terms (eg, pre bronchodilation) but not post-treatment.
  - We excluded terms that did not share the same parent term as the accepted term in the Read v2 hierarchy (compared using the first three characters of the Read v2 term), for example “662L.00 24 h blood pressure monitoring” was excluded from the blood pressure phenotype where the accepted term was “246.00 O/E blood pressure reading”.
4. Using terminology term mappings from the NHS Technology Reference data Update Distribution (TRUD) resource, we mapped non-excluded Read v2 terms to Clinical Terminology Version 3 (CTV3) terms. We only used mappings where the “IS_ASSURED” flag was set to true and included preferred and synonym terms (resulting in some cases in one-to-many maps).
5. We translated the unified list of Read v2 and CTV3 terms into SQL and extracted measurements for all biomarkers across the four data providers iteratively (Supplementary Figure S1).

### Expert review

The selection and review of codes was done by a group of clinicians with expertise spanning UK primary care and/or secondary care. Clinicians reviewed the final set of Read V2 and CTV3 terms marked for inclusion and exclusion by the algorithm and revised the set by manually including and excluding terms. Terms which were irrelevant to the biomarker phenotype of interest were marked for exclusion by experts. For example, the initial set of terms related to the forced expiratory volume in 1 s (FEV1) phenotype included multiple terms (eg, “339O100 Forced expired volume in one second/vital capacity ratio”) for FEV1/forced vital capacity (FVC) ratio measurements or predicted FEV1 measurements (eg, “339S.00 Percent predicted FEV1”) which were flagged for removal. Based on clinical experience, experts included diagnosis terms which could be used to record biomarker measurements. For example, clinical review included eosinopenia diagnosis (eg, “42K2.00 Eosinopenia”) in the eosinophil phenotype, basophilia diagnoses (eg, “42L2.00 Basophilia”) in the basophil phenotype, and weight monitoring terms such as “1622.00 Weight increasing” in the weight phenotype.

### Statistical analyses

We generated and reported descriptive statistics (mean, median, interquartile range) for extracted biomarker values stratified by provider and plotted the distribution of values using box plots. We compared the distribution of values between data providers for inconsistencies related to data recording. We calculated the sensitivity and specificity values of the algorithm though expert review by clinicians. Terms which were included in the final phenotype definition after clinician review were considered True Positives (TP), terms incorrectly excluded from the phenotype by our approach were considered False Negatives (FN) and conversely terms incorrectly included were considered as False Positives (FP). Based on the FN and FP figures we calculated the sensitivity and specificity of the algorithm for each set of terms associated with a biomarker.

For each biomarker, we fitted a Cox proportional hazards model with all-cause mortality as the outcome of interest, adjusted for sex and modeled using restricted cubic splines. We report hazard ratios from the sex-adjusted model with 95% confidence intervals.

All analyses were performed using Python v3.7 and the pandas data analysis library (v. 1.0.3, available at https://pandas.pydata.org/). Units were processed using the quantulum3 Python library (v. 0.7.3 available at https://pypi.org/project/quantulum3/).

### Data availability

Unit conversions and mappings, entity type to UKB field mappings, and lists of Read v2 and CTV3 are provided in the Appendix and online https://github.com/spiros/ukb-biomarker-phenotypes.

The Read v2 to CTV3 mapping file is available from the NHS TRUD service online https://isd.digital.nhs.uk/trud3/user/guest/group/0/home. UKB data can be obtained following approval by applying to the UKB Access Management Committee https://bbams.ndph.ox.ac.uk/ams/. Data in this project were analyzed under protocol ref. 9922 which has been approved by the UKB.

## RESULTS

### Semi-supervised identification of relevant terms from clinical terminologies

Using the algorithm described previously, we initially identified 1651 Read v2 and CTV3 terms of which 1228 were automatically excluded. The majority of terms which were automatically excluded by the algorithm was due being marked as “semantically distant”, that is they did not share a parent term with the most frequently used term for that particular phenotype. Moreover, we processed 101 raw unit values recorded in the Scottish data and mapped them to 53 harmonized values. We additionally mapped Vision-specific lookup codes for units to standardized unit definitions (eg, MEA156 maps to *mmol*). Units were not systematically recorded across the biomarkers with variable levels of missingness: systolic and diastolic blood pressure had units missing in 78% records while FEV1 had units missing in 49%. In contrast, basophils, lymphocytes, monocytes, and eosinophils had units recorded for 95% of measurements.

### Algorithm accuracy evaluation using clinical experts

**Figure 2.**
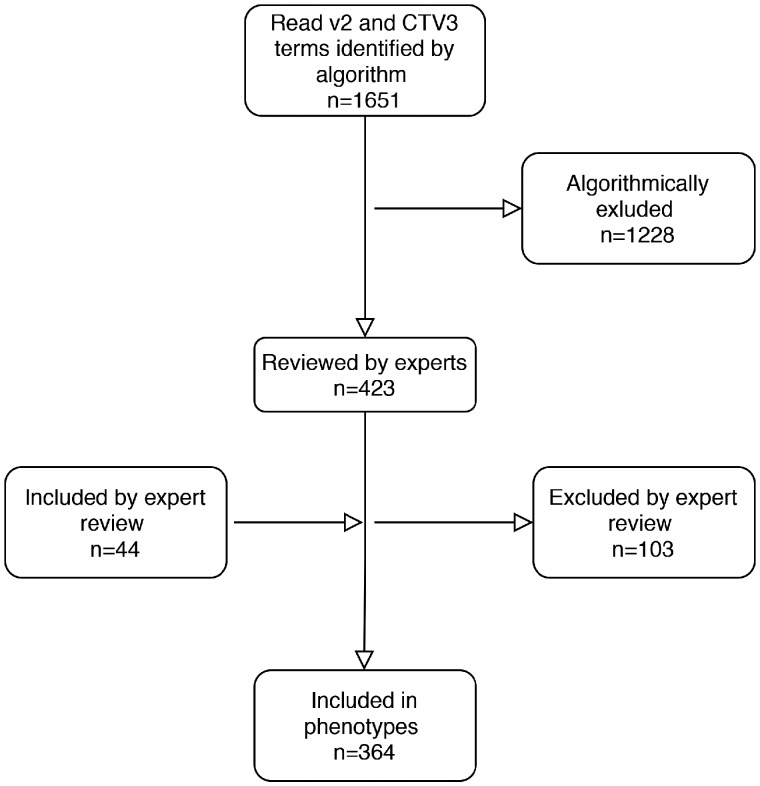
Flow diagram showing the number of Read v2 and CTV3 terms identified by the algorithm and subsequent inclusions and exclusions performed through expert review. CTV3: Clinical Terms Version 3.

Clinical experts reviewed the lists of terms for the phenotypes and manually included terms which were incorrectly flagged for exclusion (false negatives) and conversely removed terms which were incorrectly marked for inclusion (false positives). Specifically, 44 additional terms were manually included and 103 terms that had been retained by algorithm were excluded across all phenotypes. This resulted in a final set of 364 unique Read terms, which were used to extract data (Figure 2). The overall sensitivity of our approach was 0.89, while the overall specificity was 0.92. We calculated sensitivity and specificity estimates for each phenotype and report these in Supplementary Table S2. In summary, we observed the lowest sensitivity (0.6), that is the highest number of Read terms incorrectly excluded by the algorithm (false negatives) in the glucose phenotype. This was due to the fact that Read v2 terms used to record values did not share a common parent term and were distributed across different branches of the ontology, for example “*44g.00 Plasma glucose level*” and “*44TA.00 Plasma glucose*”. We observed the lowest specificity estimate (false positives) (0.66) in the red blood cell phenotype where terms related to nucleated red blood cell measurements were included. A similar pattern in terms of specificity was also observed in the FEV1 where the initial pool included terms for predicted/expected measurements, post bronchodilation values or terms related to other relevant but not exact measurements (eg, FVC).

### Exploratory analysis of the 31 biomarker phenotypes

Using the final set of Read codes, we extracted 38 190 682 events from the GP clinical events table. Of those, 34 578 209 events had a valid measurement attached to them (ie, not missing and within the valid range). Specifically, we extracted 3 616 003 measurements from England Vision (data provider 1), 1 975 448 measurements from Scotland (data provider 2), 25 233 653 measurements form England TPP (data provider 3), and 3 753 105 measurements from Wales (data provider 4). Approximately 99.5% of the participants where primary care EHR data were available had at least one biomarker measurement (*n* = 220 981, 52% female). Systolic and diastolic blood pressure were the most commonly recorded biomarkers with 3 824 851 and 4 002 384 measurements, respectively. The least often recorded marker was hematocrit percentage with 27 229 values recorded across all data sources.

**Figure 3.**
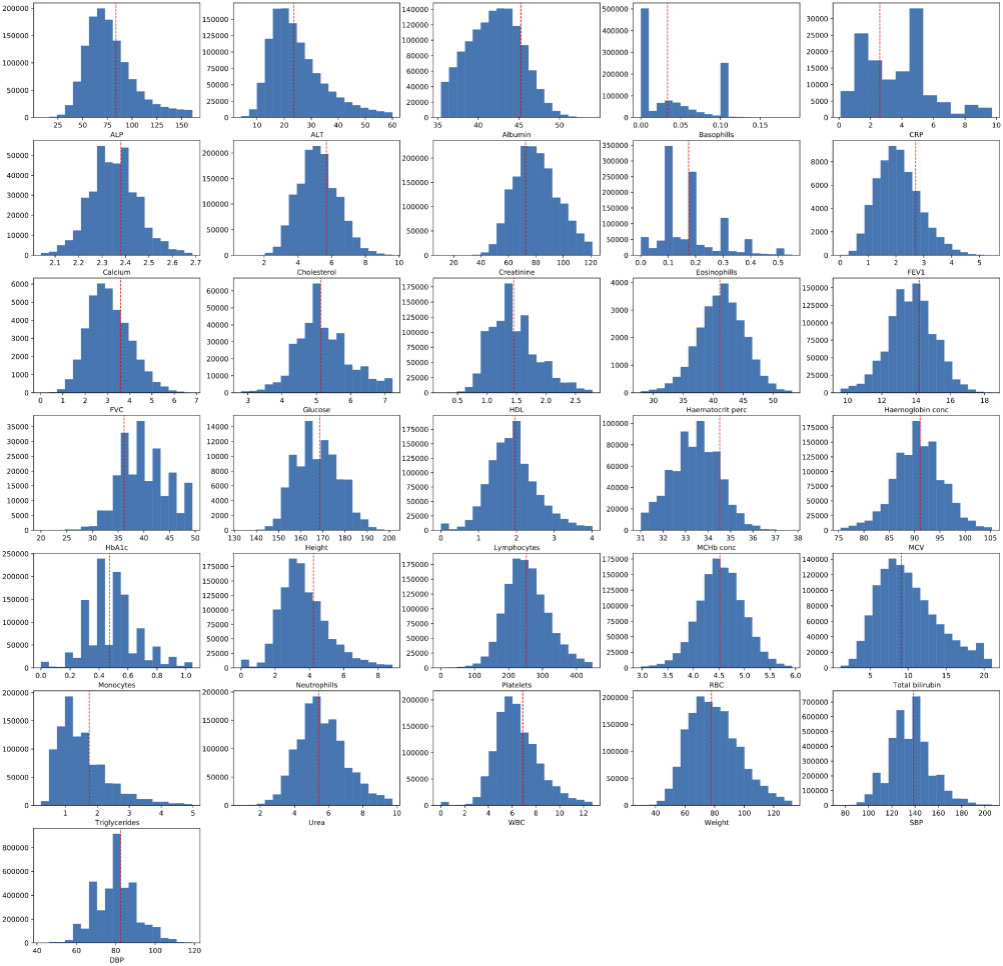
Histogram plots showing the distribution of values extracted from primary care EHR for the clinical biomarkers defined in this study. The dashed red line represents the mean value of the biomarker when measured at baseline (across any of the three waves) in study participants (value extracted from the UK Biobank Showcase). Minimum and maximum graph values have been aligned to those reported on the baseline measurements. ALP: alanine aminotransferase level; ALP: alkaline phosphatase level; CRP: C-reactive protein; DBP: diastolic blood pressure; FEV1: forced expiratory volume in 1 second; FVC: full vital capacity; HDL: high-density lipoprotein; MChb conc: mean corpuscular hemoglobin concentration; MCV: mean corpuscular volume; RBC: red blood cell; SBP: systolic blood pressure; WBC: white blood cell.

**Figure 4.**
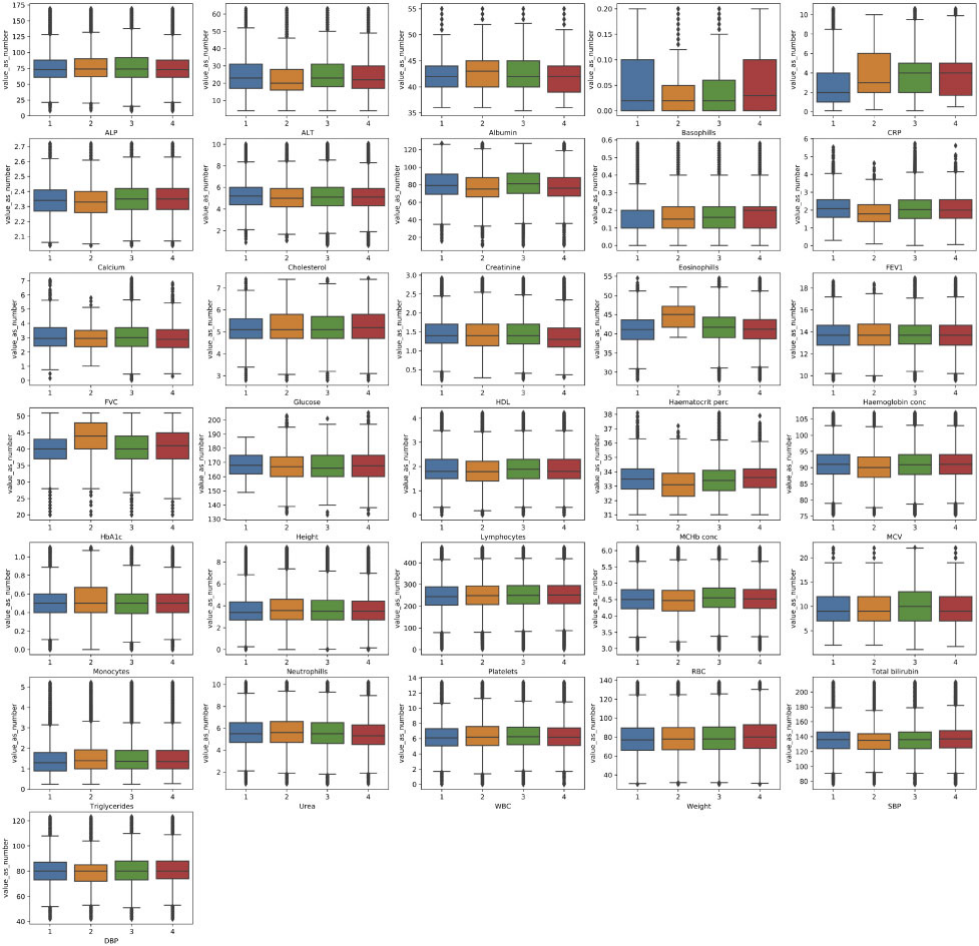
Boxplot showing the distribution of values extracted from primary care EHR for the clinical biomarkers defined in this study. 1 = England Vision, 2 = Scotland EMIS and Vision, 3 = England TPP and 4 = Wales. Minimum and maximum graph values have been aligned to those reported on the baseline measurements. ALP: alanine aminotransferase level; ALP: alkaline phosphatase level; CRP: C-reactive protein; DBP: diastolic blood pressure; FEV1: forced expiratory volume in 1 second; FVC: full vital capacity; HDL: high-density lipoprotein; MChb conc: mean corpuscular hemoglobin concentration; MCV: mean corpuscular volume; RBC: red blood cell; SBP: systolic blood pressure; WBC: white blood cell.

**Figure 5.**
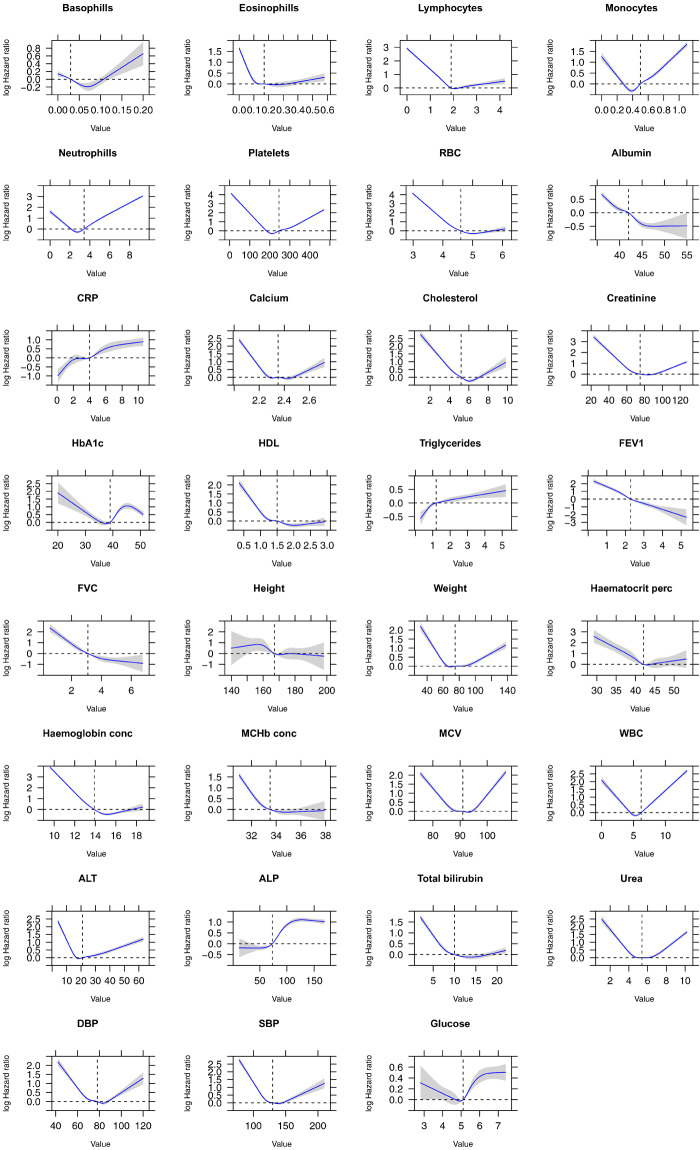
Adjusted Cox proportional hazards regression restricted cubic spline models for all biomarkers and all-cause mortality. Analyses were adjusted for patient sex and age. In each panel, the blue line indicates the estimated HR and the gray shading denotes the 95% confidence limits. The horizontal dashed line corresponds to the normal reference hazard ratio of 1.0, values above are associated with increased mortality risk, and values below are associated with decreased mortality risk compared with the reference value. ALP: alanine aminotransferase level; ALP: alkaline phosphatase level; CRP: C-reactive protein; DBP: diastolic blood pressure; FEV1: forced expiratory volume in 1 second; FVC: full vital capacity; HDL: high-density lipoprotein; MChb conc: mean corpuscular hemoglobin concentration; MCV: mean corpuscular volume; RBC: red blood cell; SBP: systolic blood pressure; WBC: white blood cell.

The distributions of each biomarker across all data sources and for each data source individually are presented in Figures 3 and 4, respectively. The observed distribution of values for each phenotype were noted to be broadly similar across all four primary care data sources as shown in Table 3. Finally, a series of Cox proportional hazards regression models, with the target of all-cause mortality, using restricted cubic splines and adjusted for sex and age were produced for each biomarker (Figure 5). The plots illustrate that most biochemical and hematological markers are associated with a U-shaped mortality-risk, with increasing risk at both extremes (ie, very high and very low values). However, there are notable exceptions, such as C-reactive protein (CRP), high-density lipoprotein, and alanine aminotransferase level. The anthropometric biomarkers (eg, height, weight, and lung function measurements), display a less consistent pattern in their association with mortality.

**Table 3.** Descriptive statistics (median and IQR) on the clinical biomarkers defined in this study covering blood counts, key biochemistry markers and physical measurements

Phenotype	Category	UKB id	Units	Eng. Vision events	Eng. Vision median (IQR)	Scotland events	Scotland median (IQR)	Eng. TPP events	Eng. TPP median (IQR)	Wales events	Wales median (IQR)
ALP	Blood biochemistry	30610	U/L	147431	73.00 (27.00)	65 017	74.00 (28.00)	907 659	74.00 (31.00)	169 068	73.00 (27.00)
ALT	Blood biochemistry	30620	U/L	114591	23.00 (14.00)	54 724	20.00 (12.00)	891 694	23.00 (13.00)	92 361	22.00 (13.00)
Albumin	Blood biochemistry	30600	g/L	144475	42.00 (4.00)	33 063	43.00 (5.00)	954 748	42.00 (5.00)	164 884	42.00 (5.00)
CRP	Blood biochemistry	30710	mg/L	23549	2.00 (3.00)	2510	3.00 (4.00)	89 406	4.00 (3.00)	18 104	4.00 (3.30)
Calcium	Blood biochemistry	30680	mmol/L	54796	2.34 (0.14)	7367	2.33 (0.14)	282 652	2.35 (0.14)	42 470	2.35 (0.14)
Cholesterol	Blood biochemistry	30690	mmol/L	140222	5.20 (1.60)	75 828	5.00 (1.70)	978 057	5.10 (1.70)	138 801	5.10 (1.60)
Creatinine	Blood biochemistry	30700	umol/L	164360	79.00 (23.00)	102 491	75.00 (22.00)	1 147 028	81.00 (23.00)	204 486	76.00 (21.00)
Glucose	Blood biochemistry	30740	mmol/L	41394	5.10 (0.90)	17 603	5.10 (1.10)	282 383	5.10 (1.00)	28 830	5.20 (1.10)
HDL	Blood biochemistry	30760	mmol/L	121635	1.40 (0.50)	46 453	1.40 (0.57)	764 837	1.40 (0.52)	92 854	1.30 (0.50)
HbA1c	Blood biochemistry	30750	mmol/mol	33799	40.00 (6.00)	5915	44.00 (8.00)	175 995	40.00 (7.00)	21 659	41.00 (9.00)
Total bilirubin	Blood biochemistry	30840	umol/L	137775	9.00 (5.00)	64 802	9.00 (5.00)	903 641	10.00 (6.00)	140 003	9.00 (5.00)
Triglycerides	Blood biochemistry	30870	mmol/L	116308	1.30 (0.90)	40 272	1.40 (0.93)	717 562	1.37 (0.90)	128 464	1.36 (0.90)
Urea	Blood biochemistry	30670	mmol/L	148897	5.50 (1.80)	75 630	5.60 (1.90)	1 022 366	5.50 (1.90)	58 332	5.30 (1.80)
Basophills	Blood count	30160	10^9/L	129939	0.02 (0.10)	53 759	0.02 (0.05)	832 814	0.02 (0.06)	138 889	0.03 (0.10)
Eosinophills	Blood count	30150	10^9/L	130840	0.20 (0.10)	52 681	0.15 (0.12)	828 186	0.16 (0.12)	142 025	0.20 (0.12)
Hematocrit perc	Blood count	30030	%	4055	41.10 (5.10)	11	45.00 (5.45)	11 971	41.70 (5.30)	11 192	41.20 (5.00)
Hemoglobin conc	Blood count	30020	g/dL	78815	13.70 (1.80)	21 512	13.60 (1.90)	903 286	13.70 (1.70)	92 144	13.70 (1.80)
Lymphocytes	Blood count	30120	10^9/L	133853	1.80 (0.80)	50 032	1.79 (0.82)	842 332	1.89 (0.80)	142 637	1.80 (0.80)
MCHb conc	Blood count	30060	g/dL	64138	33.50 (1.40)	28 062	33.50 (1.70)	607 386	33.40 (1.40)	33 023	33.60 (1.30)
MCV	Blood count	30040	fL	136768	91.00 (6.00)	54 055	90.00 (6.30)	872 756	90.90 (6.10)	147 161	91.00 (6.00)
Monocytes	Blood count	30130	10^9/L	133696	0.50 (0.20)	52 727	0.50 (0.27)	839 930	0.50 (0.21)	141 545	0.50 (0.20)
Neutrophils	Blood count	30140	10^9/L	133588	3.40 (1.65)	53 374	3.57 (1.86)	845 892	3.50 (1.79)	143 045	3.50 (1.71)
Platelets	Blood count	30080	10^9/L	137935	244.00 (84.00)	55 317	249.00 (85.00)	879 866	250.00 (84.00)	145 591	251.00 (83.00)
RBC	Blood count	30010	10^12/L	135140	4.50 (0.58)	54 030	4.47 (0.63)	869 832	4.55 (0.59)	146 016	4.52 (0.58)
WBC	Blood count	30000	10^9/L	140068	6.10 (2.25)	55 830	6.20 (2.50)	892 739	6.25 (2.30)	147 751	6.20 (2.30)
DBP	Physical measures	4079	mmHg	357987	80.00 (14.00)	393 765	80.00 (13.00)	2 833 375	80.00 (15.00)	417 257	80.00 (14.00)
FEV1	Physical measures	3063	L	6238	2.08 (0.99)	1430	1.79 (0.95)	47 847	2.03 (1.04)	6214	2.00 (1.04)
FVC	Physical measures	3062	L	2792	2.95 (1.29)	233	2.96 (1.15)	32 277	3.00 (1.31)	3778	2.89 (1.26)
Height	Physical measures	50	cm	144	168.00 (13.12)	63 262	167.00 (14.00)	1069	166.00 (15.00)	27 449	167.64 (14.98)
SBP	Physical measures	4080	mmHg	358071	136.00 (22.00)	212 521	135.00 (21.00)	2 836 175	136.00 (22.00)	418 084	137.00 (23.00)
Weight	Physical measures	21002	Kg	142704	77.00 (23.50)	181 172	77.90 (23.32)	1 137 892	78.00 (23.50)	148 988	80.00 (25.00)

*Note*: Statistics were stratified by data provider: 1 = England Vision, 2 = Scotland EMIS and Vision, 3 = England TPP and 4 = Wales.

ALP: alanine aminotransferase level; ALP: alkaline phosphatase level; CRP: C-reactive protein; DBP: diastolic blood pressure; FEV1: forced expiratory volume in 1 second; FVC: full vital capacity; HDL: high-density lipoprotein; MChb conc: mean corpuscular hemoglobin concentration; MCV: mean corpuscular volume; RBC: red blood cell; SBP: systolic blood pressure; WBC: white blood cell.

## DISCUSSION

In this study, we described a semi-supervised phenotyping approach and applied it on primary care EHR sourced from four different providers in three countries made available for UK Biobank participants. We applied our approach to produce 31 rule-based phenotyping algorithms for commonly used biomarkers with an overall sensitivity of 0.89 and specificity of 0.92. To our knowledge, this is the first study describing how phenotyping algorithms for common biomarkers can be implemented in primary care EHR for UK Biobank participants in a robust and semi-automated manner at scale.

### Association of phenotype values with all-cause mortality

In line with our phenotyping methodology,^24^ we evaluated the phenotyping algorithms created by our approach by estimating hazard ratios adjusted for age and sex with all-cause mortality and comparing our findings with known epidemiological associations. We observed similar mortality patterns with previous literature using data extracted from EHR. For example, in line with previous research, we observed an increased risk of mortality in patients with low eosinophil and low lymphocyte counts^25^ Similarly, we observed a “U” shape relationship for systolic and diastolic blood pressure measurements which is concordant to previous findings.^26^ Finally, intuitively, we observed that a decrease in FEV1 and FVC was associated with an increased risk of mortality and conversely an increase in CRP was associated with an increased risk.

### Strengths and limitations

Our method has several strengths. Firstly, it enables the rapid bootstrapping of phenotyping algorithms by reducing the number of Read terms requiring manual review by several orders of magnitude, thereby reducing the amount of resources required. Second, the approach is potentially applicable to non-UK data that face similar challenges, for example large biobanked efforts in the US such as MVP and others. Lastly, it provides research-ready phenotyping algorithms for commonly recorded biomarkers in primary care for UK Biobank users. The observed distribution values of the measurements across all biomarkers are consistent with the standard reference ranges for normal results.^27^ As previously reported^28^ UKB participants are healthier and of higher socioeconomic status than the general population so we would expect to observe these patterns in the measurements.

Our approach also has limitations. Firstly, given that the initial set of codes is from Read 2 and CTV3 terms are identified through a forward cross-map, it’s possible to omit terms that only exist in CTV3 and are used to record information. The likelihood of this happening however is low given that CTV3 encapsulates Read v2 and GPs tend to use the same set of terms over time. This has implications on the sensitivity and specificity measures reported as they refer to the values in relation to our approach identifying the correct terms to include in the algorithm (True Positives) rather than an independent “gold” standard. Due to the manner in which codes are used, similar but distinct measurements were sometimes grouped under the same entity code and were incorrectly included by the approach—for example, most lipid measurements had both plasma and serum related terms and manual review subsequently removed the plasma measurements for consistency. Similar patterns were observed, but at much lower frequency, in between fasting and random measurements and values corrected/uncorrected values were reported. Physiological measurements which are performed during routine consultations and not explicitly ordered, such as height, weight, and blood pressure required manual phenotyping given the heterogeneity in how data sources captured them despite the small number of Read terms composing the phenotypes (Supplementary Figures S2 and S3). Finally, the phenotypes created and evaluated in this manuscript are predominantly laboratory values for which a smaller set of diagnosis terms exists in terminology systems compared with disease or syndrome phenotypes which are often represented by hundreds of terms. As such, while the method described in our manuscript yields robust results for the phenotype use-cases presented here, further research is required to explore and evaluate its performance in creating phenotyping algorithms ascertaining disease status.

### Implications for researchers and policymakers

Creating phenotyping algorithms for primary care EHR, especially when they are from different data sources can be a time-consuming effort requiring a significant amount of labor and resources as there are ∼497 000 potential terms across Read version 2 and CTV3 which are inconsistently used to record information. Moreover, differences in data schemas mean that information is recorded in different ways: for example, data in Scotland can have the units specified as free text in addition to another two values while data from England (TPP) do not specify units and only cover a single value field. However, whilst measurements with different protocols (eg, BP standing or lying) may be recorded with different Read codes, importantly, they all have the same entity type. The underlying theme of our work is that our approach is a robust starting point for aggregating Read terms that may be used to record a particular clinical measurement into a meaningful biomarker phenotype. Entity types are easier to manipulate given that only a few hundred exist and the group of related Read terms can then be used to identify equivalent terms in CTV3 via the mapping and hence identify equivalent data in the different data sources. This project illustrates that the efficaciousness of the described approach, and should ideally inform future research, independent of whether the specific algorithms we have made available are used.

## CONCLUSION

In this article, we have demonstrated the challenges that UK Biobank researchers will face when extracting biomarker values from the primary care EHR records of participants. We presented a semi-supervised approach that uses existing phenotyping algorithms and semantic mappings to bootstrap algorithms for 31 common biomarkers spanning hematological and physiological measurements which are widely used in research. Our research findings are applicable to international audiences given that the controlled clinical terminologies used in the UK primary care EHR are part of SNOMED-CT, two-thirds of UK Biobank users US-based investigators and similar large-scale initiatives (eg, eMERGE, MVP) are likely to face similar challenges. As such, the phenotyping algorithms that have resulted from this work should hopefully facilitate rapid and robust access to the primary care EHR data for UKB participants during the COVID-19 public health emergency, and long after.

## FUNDING

This work was supported by the BigData@Heart program that has received funding from the Innovative Medicines Initiative 2 Joint Undertaking under grant agreement no. 116074. This Joint Undertaking receives support from the European Union’s Horizon 2020 research and innovation program and EFPIA.

This work was supported by the Health Data Research UK, which receives its funding from Health Data Research UK Ltd (LON1) funded by the UK Medical Research Council, Engineering and Physical Sciences Research Council, Economic and Social Research Council, Department of Health and Social Care (England), Chief Scientist Office of the Scottish Government Health and Social Care Directorates, Health and Social Care Research and Development Division (Welsh Government), Public Health Agency (Northern Ireland), British Heart Foundation, and the Wellcome Trust.

This work was supported by the National Institute for Health Research Biomedical Research Centre at University College London Hospitals. HH is supported by a National Institute for Health Research Senior Investigator award. ADS is supported by a THIS Institute postdoctoral fellowship. VK is supported by the Wellcome Trust (WT 110284/Z/15/Z). SD is supported by an Alan Turing Fellowship. GF is funded by the American Heart Association Institutional Data Fellowship Program (AHA Award 17IF3389000).

## AUTHOR CONTRIBUTIONS

SD conceived and designed the study, implemented the algorithm and extracted the data. AS, BM, VK, JQ reviewed and refined the phenotyping algorithm. SD analyzed the data and wrote the report, BM made substantial revisions to the report. All authors reviewed and interpreted the results, commented on the report, contributed to revisions, and read and approved the final version.

## SUPPLEMENTARY MATERIAL

Supplementary material is available at *Journal of the American Medical Informatics Association* online.

## Supplementary Material

ooaa047_Supplementary_Data
