# Supplementary material for: A semi-supervised approach for rapidly creating clinical biomarker phenotypes in the UK Biobank using different primary care EHR and clinical terminology systems
Source: JAMIA Open. 2020 Dec 5;3(4):545–56. doi: 10.1093/jamiaopen/ooaa047 (PMC7717266; doi:10.1093/jamiaopen/ooaa047)
Supplement: ooaa047_Supplementary_Data [file ooaa047_supplementary_data.docx]

**Supplementary Figure 1: Flowchart description of the main steps involved in data extraction for each biomarker. In England and Wales data sources, measurements are recorded in the *value1* field whereas in Scotland measurements are recoded in *value2* and units in *value3*.**

**
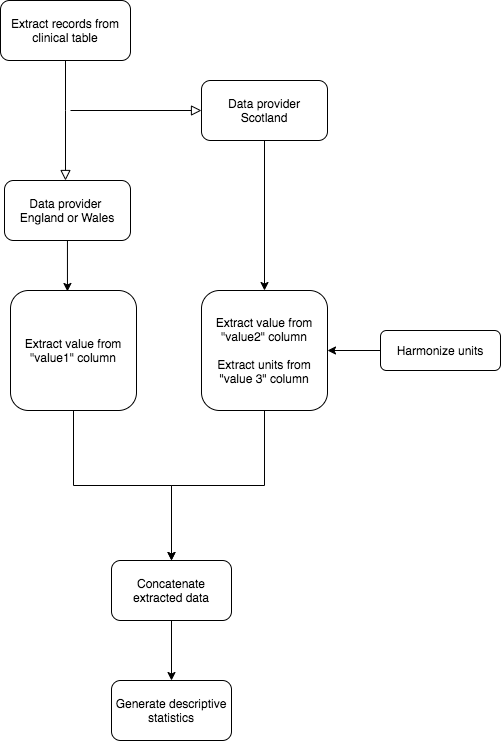
**

NB. This is a low-resolution version – a high resolution version is attached to the email.

**Supplementary Figure 2 Blood pressure phenotype pseudocode. Systolic and diastolic blood pressure are recorded differently across the four different data providers.** English data (from Vision and TPP) use the structured data fields (value1 and value2) with a non-specific Read code “246..00 O/E - blood pressure reading”' to differentiate between systolic and diastolic. Data from Scotland and Wales use a combination of both measurement specific Read codes i.e. “2469.00 O/E - Systolic BP reading” and generic terms combined with structured data fields to capture information.

IF data_provider = England Vision (1)

IF read_2 = “246..00 O/E - blood pressure reading”'

SBP = value1

DBP = value2

ELSE IF data_provider = Scotland (2)

IF read_2 = “246..00 O/E - blood pressure reading”

DBP = value1

SBP = value2

ELSE IF read_2 = “2469.00 O/E - Systolic BP reading”

SBP = value1

ELSE IF read_2 = “246A.00 O/E - Diastolic BP reading”

DBP = value1

IF data_provider = England TPP (3)

IF read_3 = “2469.00 O/E - Systolic BP reading”

SBP = value1

ELSE IF read_3 = “246A.00 O/E - Diastolic BP reading”

DBP = value1

IF data_provider = Wales (4)

IF read_2 = “246..00 O/E - blood pressure reading”

SBP = value1

DBP = value2

ELSE IF read_2 = “2469.00 O/E - Systolic BP reading”

SBP = value1

ELSE IF read_2 = “246A.00 O/E - Diastolic BP reading”

DBP = value1

**Supplementary Figure 3 Height and Weight phenotype pseudocode. Data from Scotland (data provider 2) store the height and weight values in the “value1” field as opposed to the “value2” field which is used for all other phenotypes. For the Weight phenotype, the value3 field contains the BMI of the patient (or in other cases contains the units of measurement).**

# Weight

IF read_2 in phenotype codes

IF data_provider = England Vision (1) OR data provider = Wales (4)

Weight = value1

ELSE IF data_provider = Scotland (2)

IF read_2 = '22A.. O/E - weight'

Weight = value2

ELSE IF read_3 in phenotype codes

IF data_provider = England TPP (3)

Weight = value1

# Height

IF data_provider = England Vision (1) OR data provider = Wales (4)

IF read_2 in phenotype codes

Height = value1

ELSE IF data_provider = Scotland (2)

IF read_2 = “229.. O/E – height”

Height = value1

ELSE IF data_provider = England TPP (3)

IF CTV3 code = read_3

Height = value1

**Supplementary Table 1: sensitivity and specificity of the algorithm for including and excluding Read terms from version 2 and version 3 in each phenotype following expert review.** HDL = high-density lipoprotein, ALP = alkaline phosphatase level, ALP = alanine aminotransferase level, SBP = Systolic blood pressure, DBP = diastolic blood pressure, WBC = White Blood Cell, RBC = red blood cell, CRP = C-reactive protein, MCV = Mean corpuscular volume, MChb conc = Mean corpuscular haemoglobin concentration, FEV1 = Forced Expiratory Volume in 1 second, FVC = Full Vital Capacity

| **phenotype** | **All terms** | **Algorithm included** | **Algorithm excluded** | **Final included** | **Final excluded** | **Expert included** | **Expert excluded** | **Sensitivity** | **specificity** |
| --- | --- | --- | --- | --- | --- | --- | --- | --- | --- |
| ALP | 58 | 19 | 39 | 11 | 47 | 0 | 8 | 1 | 0.85 |
| ALT | 21 | 12 | 9 | 11 | 10 | 0 | 1 | 1 | 0.90 |
| Albumin | 12 | 7 | 5 | 7 | 5 | 0 | 0 | 1 | 1 |
| Basophills | 10 | 6 | 4 | 8 | 2 | 2 | 0 | 0.8 | 1 |
| CRP | 38 | 6 | 32 | 6 | 32 | 0 | 0 | 1 | 1 |
| Calcium | 17 | 11 | 6 | 7 | 10 | 1 | 5 | 0.87 | 0.66 |
| Cholesterol | 93 | 28 | 65 | 21 | 72 | 0 | 7 | 1 | 0.91 |
| Creatinine | 42 | 17 | 25 | 17 | 25 | 0 | 0 | 1 | 1 |
| Eosinophills | 11 | 8 | 3 | 9 | 2 | 1 | 0 | 0.9 | 1 |
| FEV1 | 65 | 26 | 39 | 7 | 58 | 0 | 19 | 1 | 0.75 |
| FVC | 50 | 16 | 34 | 7 | 43 | 1 | 10 | 0.87 | 0.81 |
| Glucose | 130 | 10 | 120 | 16 | 114 | 12 | 6 | 0.57 | 0.95 |
| HDL | 28 | 8 | 20 | 6 | 22 | 0 | 2 | 1 | 0.916667 |
| Haematocrit perc | 19 | 14 | 5 | 18 | 1 | 4 | 0 | 0.81 | 1 |
| Haemoglobin conc | 61 | 25 | 36 | 22 | 39 | 2 | 5 | 0.91 | 0.88 |
| HbA1c | 49 | 18 | 31 | 25 | 24 | 7 | 0 | 0.78 | 1 |
| Height | 229 | 6 | 223 | 6 | 223 | 0 | 0 | 1 | 1 |
| Lymphocytes | 31 | 15 | 16 | 7 | 24 | 0 | 8 | 1 | 0.75 |
| MCHb conc | 10 | 6 | 4 | 10 | 0 | 4 | 0 | 0.71 | 0 |
| MCV | 17 | 11 | 6 | 13 | 4 | 2 | 0 | 0.86 | 1 |
| Monocytes | 17 | 12 | 5 | 9 | 8 | 1 | 4 | 0.9 | 0.66 |
| Neutrophills | 20 | 13 | 7 | 13 | 7 | 0 | 0 | 1 | 1 |
| Platelets | 34 | 17 | 17 | 12 | 22 | 0 | 5 | 1 | 0.81 |
| RBC | 16 | 10 | 6 | 12 | 4 | 4 | 2 | 0.75 | 0.66 |
| Total bilirubin | 37 | 24 | 13 | 15 | 22 | 0 | 9 | 1 | 0.70 |
| Triglycerides | 32 | 15 | 17 | 15 | 17 | 0 | 0 | 1 | 1 |
| Urea | 47 | 24 | 23 | 17 | 30 | 0 | 7 | 1 | 0.81 |
| WBC | 49 | 22 | 27 | 19 | 30 | 0 | 3 | 1 | 0.90 |
| Weight | 408 | 17 | 391 | 18 | 390 | 3 | 2 | 0.85 | 0.99 |

**Supplementary Table 2: Read codes (Read v2 and CTV3) included in the phenotyping algorithms.** HDL = high-density lipoprotein, ALP = alkaline phosphatase level, ALP = alanine aminotransferase level, SBP = Systolic blood pressure, DBP = diastolic blood pressure, WBC = White Blood Cell, RBC = red blood cell, CRP = C-reactive protein, MCV = Mean corpuscular volume, MChb conc = Mean corpuscular haemoglobin concentration, FEV1 = Forced Expiratory Volume in 1 second, FVC = Full Vital Capacity

| phenotype | terminology | readcode | readterm |
| --- | --- | --- | --- |
| ALP | read2 | 44F..00 | Serum alkaline phosphatase |
| ALP | read2 | 44F3.00 | Total alkaline phosphatase |
| ALP | read2 | 44FZ.00 | Serum alkaline phosphatase NOS |
| ALP | read2 | 44F1.00 | Serum alk. phos. normal |
| ALP | read2 | 44F2.00 | Serum alk. phos. raised |
| ALP | ctv3 | XE2px | Serum alkaline phosphatase |
| ALP | ctv3 | XE2px | Serum alkaline phosphatase level |
| ALP | ctv3 | 44F3. | Alkaline phosphatase level |
| ALP | ctv3 | 44FZ. | Serum alkaline phosphatase NOS |
| ALP | ctv3 | 44F1. | Serum alk. phos. normal |
| ALP | ctv3 | 44F2. | Serum alk. phos. raised |
| ALT | read2 | 44G3.00 | ALT/SGPT serum level |
| ALT | read2 | 44GB.00 | Serum alanine aminotransferase level |
| ALT | read2 | 44G..11 | ALT - blood level |
| ALT | read2 | 44G3000 | ALT/SGPT level normal |
| ALT | read2 | 44G3100 | ALT/SGPT level abnormal |
| ALT | ctv3 | 44G3. | ALT/SGPT serum level |
| ALT | ctv3 | XaLJx | Serum alanine aminotransferase level |
| ALT | ctv3 | X771f | ALT - blood level |
| ALT | ctv3 | X771e | SGPT - blood level |
| ALT | ctv3 | 44G30 | ALT.SGPT level normal |
| ALT | ctv3 | 44G31 | ALT.SGPT level abnormal |
| Albumin | read2 | 44M4.00 | Serum albumin |
| Albumin | read2 | 44M4000 | Serum albumin normal |
| Albumin | read2 | 44M4100 | Serum albumin low |
| Albumin | ctv3 | XE2eA | Serum albumin |
| Albumin | ctv3 | XE2eA | Serum albumin level |
| Albumin | ctv3 | 44M40 | Serum albumin normal |
| Albumin | ctv3 | 44M41 | Serum albumin low |
| Basophills | read2 | 42L..00 | Basophil count |
| Basophills | read2 | 42LZ.00 | Basophil count NOS |
| Basophills | read2 | 42L1.00 | Basophil count normal |
| Basophills | ctv3 | 42L.. | Basophil count |
| Basophills | ctv3 | 42LZ. | Basophil count NOS |
| Basophills | ctv3 | 42L1. | Basophil count normal |
| Basophills | read2 | 42L3.00 | Basophil count abnormal |
| Basophills | read2 | 42L2.00 | Basophilia |
| CRP | read2 | 44CS.00 | Serum C reactive protein level |
| CRP | read2 | 44CC000 | C reactive protein normal |
| CRP | read2 | 44CC100 | C reactive protein abnormal |
| CRP | ctv3 | XaINL | Serum C reactive protein level |
| CRP | ctv3 | 44CC0 | C-reactive protein normal |
| CRP | ctv3 | 44CC1 | C-reactive protein abnormal |
| Calcium | read2 | 44I8.00 | Serum calcium |
| Calcium | read2 | 44I8000 | Normal serum calcium level |
| Calcium | ctv3 | XE2q3 | Serum calcium |
| Calcium | ctv3 | XE2q3 | Serum calcium level |
| Calcium | ctv3 | 44I80 | Normal serum calcium level |
| Calcium | ctv3 | Xabpk | Serum adjusted calcium concentration |
| Calcium | read2 | 44h4.00 | Blood calcium level |
| Cholesterol | read2 | 44P..00 | Serum cholesterol |
| Cholesterol | read2 | 44PJ.00 | Serum total cholesterol level |
| Cholesterol | read2 | 44PH.00 | Total cholesterol measurement |
| Cholesterol | read2 | 44P3.00 | Serum cholesterol raised |
| Cholesterol | read2 | 44P1.00 | Serum cholesterol normal |
| Cholesterol | read2 | 44PZ.00 | Serum cholesterol NOS |
| Cholesterol | read2 | 44P9.00 | Serum cholesterol studies |
| Cholesterol | read2 | 44P2.00 | Serum cholesterol borderline |
| Cholesterol | read2 | 44PK.00 | Serum fasting total cholesterol |
| Cholesterol | read2 | 44P4.00 | Serum cholesterol very high |
| Cholesterol | ctv3 | XE2eD | Serum cholesterol |
| Cholesterol | ctv3 | XE2eD | Serum cholesterol level |
| Cholesterol | ctv3 | XaJe9 | Serum total cholesterol level |
| Cholesterol | ctv3 | XSK14 | Total cholesterol measurement |
| Cholesterol | ctv3 | 44P3. | Serum cholesterol raised |
| Cholesterol | ctv3 | 44P1. | Serum cholesterol normal |
| Cholesterol | ctv3 | 44PZ. | Serum cholesterol NOS |
| Cholesterol | ctv3 | 44P9. | Serum cholesterol studies |
| Cholesterol | ctv3 | 44P2. | Serum cholesterol borderline |
| Cholesterol | ctv3 | XaLux | Serum fasting total cholesterol |
| Cholesterol | ctv3 | 44P4. | Serum cholesterol very high |
| Creatinine | read2 | 44J3.00 | Serum creatinine |
| Creatinine | read2 | 44J3200 | Serum creatinine normal |
| Creatinine | read2 | 44J3z00 | Serum creatinine NOS |
| Creatinine | read2 | 44J3300 | Serum creatinine raised |
| Creatinine | read2 | 44JD.00 | Corrected serum creatinine level |
| Creatinine | read2 | 44JC.00 | Corrected plasma creatinine level |
| Creatinine | read2 | 44J3100 | Serum creatinine low |
| Creatinine | read2 | 44J3000 | Serum creatinine abnormal |
| Creatinine | ctv3 | XE2q5 | Serum creatinine |
| Creatinine | ctv3 | XE2q5 | Serum creatinine level |
| Creatinine | ctv3 | 44J32 | Serum creatinine normal |
| Creatinine | ctv3 | 44J3z | Serum creatinine NOS |
| Creatinine | ctv3 | 44J33 | Serum creatinine raised |
| Creatinine | ctv3 | XaERc | Corrected serum creatinine level |
| Creatinine | ctv3 | XaERX | Corrected plasma creatinine level |
| Creatinine | ctv3 | 44J31 | Serum creatinine low |
| Creatinine | ctv3 | 44J30 | Serum creatinine abnormal |
| Eosinophills | read2 | 42K..00 | Eosinophil count |
| Eosinophills | read2 | 42KZ.00 | Eosinophil count NOS |
| Eosinophills | read2 | 42K1.00 | Eosinophil count normal |
| Eosinophills | read2 | 42K3.00 | Eosinophil count raised |
| Eosinophills | ctv3 | 42K.. | Eosinophil count |
| Eosinophills | ctv3 | 42KZ. | Eosinophil count NOS |
| Eosinophills | ctv3 | 42K1. | Eosinophil count normal |
| Eosinophills | ctv3 | 42K3. | Eosinophil count raised |
| Eosinophills | read2 | 42K2.00 | Eosinopenia |
| FEV1 | read2 | 339O.00 | Forced expired volume in 1 second |
| FEV1 | read2 | 3397 | Forced expiratory volume - FEV |
| FEV1 | read2 | 339a.00 | FEV1 before bronchodilation |
| FEV1 | read2 | 3397000 | FEV normal |
| FEV1 | ctv3 | X77Qu | Forced expired volume in 1 second |
| FEV1 | ctv3 | XaIxQ | FEV1 before bronchodilation |
| FEV1 | ctv3 | 33970 | FEV normal |
| FVC | read2 | 3396 | Forced vital capacity - FVC |
| FVC | read2 | 3396000 | FVC - forced vital capacity normal |
| FVC | read2 | 3396100 | FVC - forced vital capacity abnormal |
| FVC | ctv3 | 3396 | Forced vital capacity |
| FVC | ctv3 | 33960 | FVC - forced vital capacity normal |
| FVC | ctv3 | 33961 | FVC - forced vital capacity abnormal |
| FVC | read2 | 339s.00 | Forced vital capacity before bronchodilation |
| Glucose | read2 | 44g..00 | Plasma glucose level |
| Glucose | read2 | 44g0.00 | Plasma random glucose level |
| Glucose | ctv3 | XM0ly | Plasma glucose level |
| Glucose | ctv3 | 44g0. | Plasma random glucose level |
| Glucose | read2 | 44U..00 | Blood glucose result |
| Glucose | read2 | 44TJ.00 | Blood glucose level |
| Glucose | read2 | 44U..11 | Blood sugar result |
| Glucose | read2 | 44TA.00 | Plasma glucose |
| Glucose | read2 | 44U4.00 | Blood glucose 5-6.9 mmol/L |
| Glucose | read2 | 44T1000 | Random blood sugar normal |
| Glucose | read2 | 44U3.00 | Blood glucose 2.5-4.9 mmol/L |
| Glucose | read2 | 44U8.00 | Blood glucose normal |
| Glucose | read2 | 44U5.00 | Blood glucose 7-9.9 mmol/L |
| Glucose | read2 | 44U..12 | Plasma glucose level |
| Glucose | read2 | 44U6.00 | Blood glucose 10-13.9 mmol/L |
| Glucose | read2 | 44T5.00 | Laboratory blood sugar |
| HDL | read2 | 44P5.00 | Serum HDL cholesterol level |
| HDL | read2 | 44PB.00 | Serum fasting HDL cholesterol level |
| HDL | read2 | 44PC.00 | Serum random HDL cholesterol level |
| HDL | ctv3 | 44P5. | Serum HDL cholesterol level |
| HDL | ctv3 | 44PB. | Serum fasting HDL cholesterol level |
| HDL | ctv3 | 44PC. | Serum random HDL cholesterol level |
| Haematocrit perc | read2 | 4258 | Haematocrit |
| Haematocrit perc | read2 | 4257 | Packed cell volume |
| Haematocrit perc | read2 | 425..00 | Haematocrit - PCV |
| Haematocrit perc | read2 | 425..11 | Packed cell volume - PCV |
| Haematocrit perc | read2 | 425Z.00 | Haematocrit - PCV - NOS |
| Haematocrit perc | read2 | 4254 | Haematocrit - PCV - low |
| Haematocrit perc | read2 | 4251 | Haematocrit - PCV - normal |
| Haematocrit perc | ctv3 | X76tb | Haematocrit |
| Haematocrit perc | ctv3 | X76tc | Packed cell volume |
| Haematocrit perc | ctv3 | XE2Zq | Haematocrit - PCV |
| Haematocrit perc | ctv3 | XE2Zq | Haematocrit - PCV level |
| Haematocrit perc | ctv3 | 425Z. | Haematocrit - PCV - NOS |
| Haematocrit perc | ctv3 | 4254 | Haematocrit - PCV - low |
| Haematocrit perc | ctv3 | 4251 | Haematocrit - PCV - normal |
| Haematocrit perc | read2 | 4255 | Haematocrit - borderline low |
| Haematocrit perc | read2 | 4253 | Haematocrit - PCV - high |
| Haematocrit perc | read2 | 4256 | Haematocrit - PCV - abnormal |
| Haematocrit perc | read2 | 4252 | Haematocrit - borderline high |
| Haemoglobin conc | read2 | 423..00 | Haemoglobin estimation |
| Haemoglobin conc | read2 | 423..11 | Hb estimation |
| Haemoglobin conc | read2 | 4237 | Haemoglobin normal |
| Haemoglobin conc | read2 | 4235 | Haemoglobin low |
| Haemoglobin conc | read2 | 423Z.00 | Haemoglobin estimation NOS |
| Haemoglobin conc | read2 | 4239 | Haemoglobin high |
| Haemoglobin conc | read2 | 4236 | Haemoglobin borderline low |
| Haemoglobin conc | read2 | 4234 | Haemoglobin very low |
| Haemoglobin conc | read2 | 423B.00 | Haemoglobin abnormal |
| Haemoglobin conc | ctv3 | Xa96v | Haemoglobin concentration |
| Haemoglobin conc | ctv3 | XE2m6 | Haemoglobin estimation |
| Haemoglobin conc | ctv3 | XM1Vu | Hb estimation |
| Haemoglobin conc | ctv3 | 4237 | Haemoglobin normal |
| Haemoglobin conc | ctv3 | 4235 | Haemoglobin low |
| Haemoglobin conc | ctv3 | 423Z. | Haemoglobin estimation NOS |
| Haemoglobin conc | ctv3 | 4239 | Haemoglobin high |
| Haemoglobin conc | ctv3 | 4236 | Haemoglobin borderline low |
| Haemoglobin conc | ctv3 | X76ti | Haemoglobin H inclusion |
| Haemoglobin conc | ctv3 | 4234 | Haemoglobin very low |
| Haemoglobin conc | ctv3 | 423B. | Haemoglobin abnormal |
| Haemoglobin conc | read2 | 4238 | Haemoglobin borderline high |
| Haemoglobin conc | read2 | 423A.00 | Haemoglobin very high |
| HbA1c | read2 | 42W5.00 | Haemoglobin A1c level - IFCC standardised |
| HbA1c | read2 | 42W4.00 | HbA1c level (DCCT aligned) |
| HbA1c | read2 | 42W..00 | Hb. A1C - diabetic control |
| HbA1c | read2 | 42W..11 | Glycosylated Hb |
| HbA1c | read2 | 42W..12 | Glycated haemoglobin |
| HbA1c | read2 | 42W2.00 | Hb. A1C 7-10% - borderline |
| HbA1c | read2 | 42W1.00 | Hb. A1C < 7% - good control |
| HbA1c | read2 | 42WZ.00 | Hb. A1C - diabetic control NOS |
| HbA1c | read2 | 42W3.00 | Hb. A1C > 10% - bad control |
| HbA1c | ctv3 | XaPbt | Haemoglobin A1c level - International Federation of Clinical Chemistry and Laboratory Medicine standardised |
| HbA1c | ctv3 | XaERp | HbA1c level (DCCT aligned) |
| HbA1c | ctv3 | XE24t | Hb. A1C - diabetic control |
| HbA1c | ctv3 | X80U3 | Glycated haemoglobin |
| HbA1c | ctv3 | X80U3 | Glycosylated Hb |
| HbA1c | ctv3 | 42W2. | Hb. A1C 7-10% - borderline |
| HbA1c | ctv3 | 42W1. | Hb. A1C < 7% - good control |
| HbA1c | ctv3 | 42WZ. | Hb. A1C - diabetic control NOS |
| HbA1c | ctv3 | 42W3. | Hb. A1C > 10% - bad control |
| HbA1c | read2 | 44TB.00 | Haemoglobin A1c level |
| HbA1c | read2 | 42c..00 | HbA1 - diabetic control |
| HbA1c | read2 | 42c3.00 | HbA1 level (DCCT aligned) |
| HbA1c | read2 | 42c1.00 | HbA1 7 - 10% - borderline control |
| HbA1c | read2 | 42c0.00 | HbA1 < 7% - good control |
| HbA1c | read2 | 44TC.00 | Haemoglobin A1 level |
| HbA1c | read2 | 42c2.00 | HbA1 > 10% - bad control |
| Height | read2 | 229..00 | O/E - height |
| Height | read2 | 229Z.00 | O/E - height NOS |
| Height | read2 | 2293 | O/E -height within 10% average |
| Height | ctv3 | 229.. | O/E - height |
| Height | ctv3 | 229Z. | O/E - height NOS |
| Height | ctv3 | 2293 | O/E -height within 10% average |
| Lymphocytes | read2 | 42M..00 | Lymphocyte count |
| Lymphocytes | read2 | 42MZ.00 | Lymphocyte count NOS |
| Lymphocytes | read2 | 42M1.00 | Lymphocyte count normal |
| Lymphocytes | read2 | 42M4.00 | Abnormal lymphocytes |
| Lymphocytes | ctv3 | 42M.. | Lymphocyte count |
| Lymphocytes | ctv3 | 42MZ. | Lymphocyte count NOS |
| Lymphocytes | ctv3 | 42M1. | Lymphocyte count normal |
| MCHb conc | read2 | 429..00 | Mean corpusc. Hb. conc. (MCHC) |
| MCHb conc | read2 | 429Z.00 | MCHC - NOS |
| MCHb conc | read2 | 4291 | MCHC - normal |
| MCHb conc | ctv3 | 429.. | Mean cell haemoglobin concentration |
| MCHb conc | ctv3 | 429Z. | MCHC - NOS |
| MCHb conc | ctv3 | 4291 | MCHC - normal |
| MCHb conc | read2 | 4294 | MCHC - raised |
| MCHb conc | read2 | 4293 | MCHC - low |
| MCHb conc | read2 | 4292 | MCHC - borderline low |
| MCHb conc | read2 | 4295 | MCHC - borderline raised |
| MCV | read2 | 42A..00 | Mean corpuscular volume (MCV) |
| MCV | read2 | 42A..11 | Mean cell volume |
| MCV | read2 | 42AZ.00 | MCV - NOS |
| MCV | read2 | 42A1.00 | MCV - normal |
| MCV | read2 | 42A3.00 | MCV - raised |
| MCV | read2 | 42A4.00 | MCV - low |
| MCV | ctv3 | 42A.. | Mean cell volume |
| MCV | ctv3 | 42AZ. | MCV - NOS |
| MCV | ctv3 | 42A1. | MCV - normal |
| MCV | ctv3 | 42A3. | MCV - raised |
| MCV | ctv3 | 42A4. | MCV - low |
| MCV | read2 | 42A2.00 | MCV - borderline raised |
| MCV | read2 | 42A5.00 | MCV - borderline low |
| Monocytes | read2 | 42N..00 | Monocyte count |
| Monocytes | read2 | 42NZ.00 | Monocyte count NOS |
| Monocytes | read2 | 42N1.00 | Monocyte count normal |
| Monocytes | read2 | 42N5.00 | Monocyte count abnormal |
| Monocytes | ctv3 | 42N.. | Monocyte count |
| Monocytes | ctv3 | 42NZ. | Monocyte count NOS |
| Monocytes | ctv3 | 42N1. | Monocyte count normal |
| Monocytes | ctv3 | 42N5. | Monocyte count abnormal |
| Monocytes | read2 | 42N2.00 | Monocyte count raised |
| Neutrophills | read2 | 42J..00 | Neutrophil count |
| Neutrophills | read2 | 42JZ.00 | Neutrophil count NOS |
| Neutrophills | read2 | 42J..11 | Granulocyte count |
| Neutrophills | read2 | 42J2.00 | Neutropenia |
| Neutrophills | read2 | 42J1.00 | Neutrophil count normal |
| Neutrophills | read2 | 42J3.00 | Neutrophilia |
| Neutrophills | read2 | 42J4.00 | Neutrophil count abnormal |
| Neutrophills | ctv3 | 42J.. | Neutrophil count |
| Neutrophills | ctv3 | 42JZ. | Neutrophil count NOS |
| Neutrophills | ctv3 | 42J2. | Neutropenia |
| Neutrophills | ctv3 | 42J1. | Neutrophil count normal |
| Neutrophills | ctv3 | 42J3. | Neutrophilia |
| Neutrophills | ctv3 | 42J4. | Neutrophil count abnormal |
| Platelets | read2 | 42P..00 | Platelet count |
| Platelets | read2 | 42P1.00 | Platelet count normal |
| Platelets | read2 | 42PZ.00 | Platelet count NOS |
| Platelets | read2 | 42P2.00 | Thrombocytopenia |
| Platelets | read2 | 42P3.00 | Thrombocythaemia |
| Platelets | read2 | 42P4.00 | Platelet count abnormal |
| Platelets | ctv3 | 42P.. | Platelet count |
| Platelets | ctv3 | 42P1. | Platelet count normal |
| Platelets | ctv3 | 42PZ. | Platelet count NOS |
| Platelets | ctv3 | XE24o | Thrombocytopenia |
| Platelets | ctv3 | 42P3. | Thrombocythaemia |
| Platelets | ctv3 | 42P4. | Platelet count abnormal |
| RBC | read2 | 426..00 | Red blood cell (RBC) count |
| RBC | read2 | 426Z.00 | RBC count NOS |
| RBC | read2 | 4261 | RBC count normal |
| RBC | read2 | 4263 | RBC count low |
| RBC | ctv3 | 426.. | Red blood cell count |
| RBC | ctv3 | 426Z. | RBC count NOS |
| RBC | ctv3 | 4261 | RBC count normal |
| RBC | ctv3 | 4263 | RBC count low |
| RBC | read2 | 4262 | RBC count borderline low |
| RBC | read2 | 4264 | RBC count raised |
| RBC | read2 | 4267 | RBC count abnormal |
| RBC | read2 | 4265 | RBC count borderline raised |
| Total bilirubin | read2 | 44EC.00 | Serum total bilirubin level |
| Total bilirubin | read2 | 44E..00 | Serum bilirubin level |
| Total bilirubin | read2 | 44E3.00 | Total bilirubin |
| Total bilirubin | read2 | 44E1.00 | Serum bilirubin normal |
| Total bilirubin | read2 | 44EZ.00 | Serum bilirubin NOS |
| Total bilirubin | read2 | 44E2.00 | Serum bilirubin raised |
| Total bilirubin | read2 | 44E6.00 | Serum bilirubin borderline |
| Total bilirubin | ctv3 | XaERu | Serum total bilirubin level |
| Total bilirubin | ctv3 | 44E.. | Serum bilirubin level |
| Total bilirubin | ctv3 | XE2qu | Total bilirubin level |
| Total bilirubin | ctv3 | XE2qu | Total bilirubin |
| Total bilirubin | ctv3 | 44E1. | Serum bilirubin normal |
| Total bilirubin | ctv3 | 44EZ. | Serum bilirubin NOS |
| Total bilirubin | ctv3 | 44E2. | Serum bilirubin raised |
| Total bilirubin | ctv3 | 44E6. | Serum bilirubin borderline |
| Triglycerides | read2 | 44Q..00 | Serum triglycerides |
| Triglycerides | read2 | 44Q4.00 | Serum fasting triglyceride level |
| Triglycerides | read2 | 44Q5.00 | Serum random triglyceride level |
| Triglycerides | read2 | 44QZ.00 | Serum triglycerides NOS |
| Triglycerides | read2 | 44Q1.00 | Serum triglycerides normal |
| Triglycerides | read2 | 44Q3.00 | Serum triglycerides raised |
| Triglycerides | read2 | 44Q2.00 | Serum triglycerides borderline |
| Triglycerides | ctv3 | XE2q9 | Serum triglycerides |
| Triglycerides | ctv3 | XE2q9 | Serum triglyceride levels |
| Triglycerides | ctv3 | 44Q4. | Serum fasting triglyceride level |
| Triglycerides | ctv3 | 44Q5. | Serum random triglyceride level |
| Triglycerides | ctv3 | 44QZ. | Serum triglycerides NOS |
| Triglycerides | ctv3 | 44Q1. | Serum triglycerides normal |
| Triglycerides | ctv3 | 44Q3. | Serum triglycerides raised |
| Triglycerides | ctv3 | 44Q2. | Serum triglycerides borderline |
| Urea | read2 | 44J9.00 | Serum urea level |
| Urea | read2 | 44J8.00 | Blood urea |
| Urea | read2 | 44J..11 | Urea - blood |
| Urea | read2 | 44JA.00 | Plasma urea level |
| Urea | read2 | 44J..00 | Blood urea/renal function |
| Urea | read2 | 44J1.00 | Blood urea normal |
| Urea | read2 | 44J2.00 | Blood urea abnormal |
| Urea | read2 | 44J..13 | Serum urea level |
| Urea | read2 | 44JZ.00 | Blood urea/renal function NOS |
| Urea | read2 | 44J8.11 | Urea - blood |
| Urea | ctv3 | XM0lt | Serum urea level |
| Urea | ctv3 | X771P | Blood urea |
| Urea | ctv3 | X771P | Urea - blood |
| Urea | ctv3 | XaDvl | Plasma urea level |
| Urea | ctv3 | 44J1. | Blood urea normal |
| Urea | ctv3 | 44J2. | Blood urea abnormal |
| Urea | ctv3 | 44JZ. | Blood urea/renal function NOS |
| WBC | read2 | 42H..00 | Total white cell count |
| WBC | read2 | 42H..11 | White blood count |
| WBC | read2 | 42H..12 | White cell count |
| WBC | read2 | 42H7.00 | Total white blood count |
| WBC | read2 | 42H1.00 | White cell count normal |
| WBC | read2 | 42HZ.00 | Total white cell count NOS |
| WBC | read2 | 42H3.00 | Leucocytosis -high white count |
| WBC | read2 | 42H8.00 | Total WBC (IMM) |
| WBC | read2 | 42H2.00 | Leucopenia - low white count |
| WBC | read2 | 42H5.00 | White cell count abnormal |
| WBC | ctv3 | XaIdY | Total white blood count |
| WBC | ctv3 | 42H.. | White blood cell count |
| WBC | ctv3 | 42H.. | White blood cell count - observation |
| WBC | ctv3 | 42H1. | White cell count normal |
| WBC | ctv3 | 42H3. | Leucocytosis |
| WBC | ctv3 | 42HZ. | Total white cell count NOS |
| WBC | ctv3 | XaIdZ | Total WBC (IMM) |
| WBC | ctv3 | 42H2. | Leucopenia |
| WBC | ctv3 | 42H5. | White cell count abnormal |
| Weight | read2 | 22A..00 | O/E - weight |
| Weight | read2 | 22AZ.00 | O/E - weight NOS |
| Weight | read2 | 22A4.00 | O/E - weight 10-20% over ideal |
| Weight | read2 | 22A3.00 | O/E - weight within 10% ideal |
| Weight | read2 | 22A5.00 | O/E - weight > 20% over ideal |
| Weight | read2 | 22A4.11 | O/E - overweight |
| Weight | read2 | 22A5.11 | O/E - obese |
| Weight | read2 | 22A6.00 | O/E - Underweight |
| Weight | ctv3 | 22A.. | O/E - weight |
| Weight | ctv3 | 22AZ. | O/E - weight NOS |
| Weight | ctv3 | XE1h3 | O/E - weight 10-20% over ideal |
| Weight | ctv3 | XM1YD | O/E - overweight |
| Weight | ctv3 | 22A3. | O/E - weight within 10% ideal |
| Weight | ctv3 | 222A. | O/E - obese |
| Weight | ctv3 | 22A6. | O/E - Underweight |
| Weight | read2 | 1622 | Weight increasing |
| Weight | read2 | 22A2.00 | O/E -weight 10-20% below ideal |
| Weight | read2 | 22A1.00 | O/E - weight > 20% below ideal |
